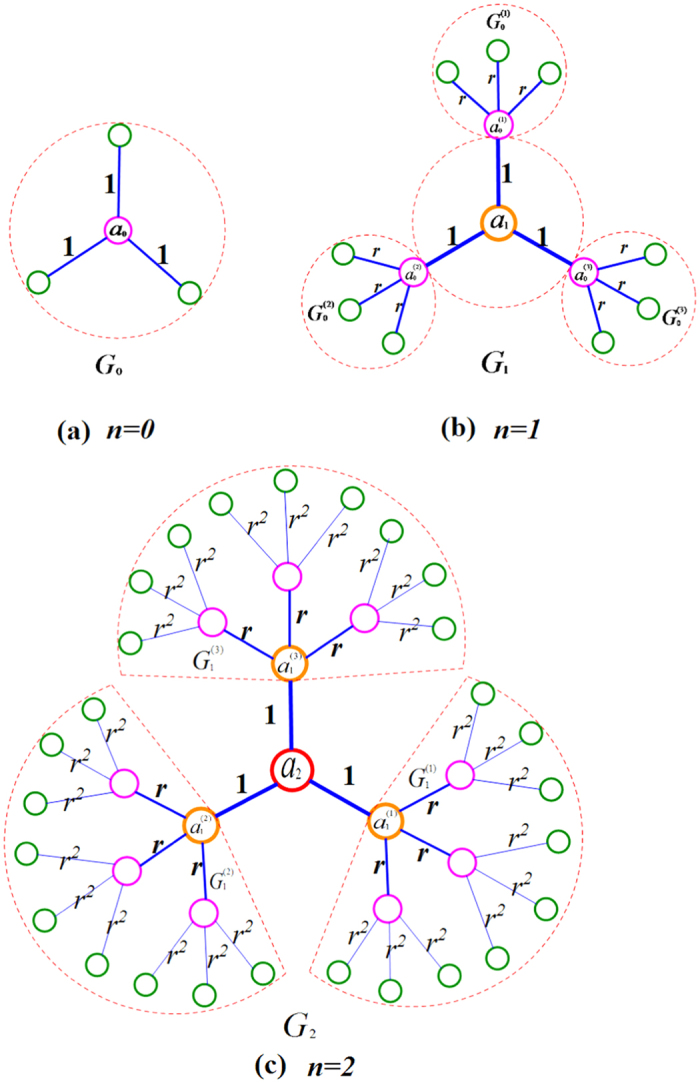# The entire mean weighted first-passage time on a family of weighted treelike networks

**DOI:** 10.1038/srep28733

**Published:** 2016-06-30

**Authors:** Meifeng Dai, Yanqiu Sun, Yu Sun, Lifeng Xi, Shuxiang Shao

**Affiliations:** 1 Nonlinear Scientific Research Center, Faculty of Science, Jiangsu University Zhenjiang, Jiangsu, 212013, P.R. China; 2 Department of Mathematics, Ningbo University, Ningbo, 315211, P.R. China

## Abstract

In this paper, we consider the entire mean weighted first-passage time (EMWFPT) with random walks on a family of weighted treelike networks. The EMWFPT on weighted networks is proposed for the first time in the literatures. The dominating terms of the EMWFPT obtained by the following two methods are coincident. On the one hand, using the construction algorithm, we calculate the receiving and sending times for the central node to obtain the asymptotic behavior of the EMWFPT. On the other hand, applying the relationship equation between the EMWFPT and the average weighted shortest path, we also obtain the asymptotic behavior of the EMWFPT. The obtained results show that the effective resistance is equal to the weighted shortest path between two nodes. And the dominating term of the EMWFPT scales linearly with network size in large network.

In recent years, the study of networks associated with complex systems has received the attention of researchers from many different areas. Especially, weighted networks^1^,^2^ represent the natural framework to describe natural, social, and technological systems^3^. The deterministic weighted networks have attracted increasing attentions because many network characteristics are exactly solved through their quantities, such as mean weighted first-passage time, average weighted shortest path^4^ etc.

Several recent works have studied the mean first-passage time (MFPT) for some self-similar weighted network models. Dai *et al*.^5^ found that the weighted Koch networks are more efficient than classic Koch networks in receiving information when a walker chooses one of its nearest neighbors with probability proportional to the weight of edge linking them (weight-dependent walk). Then Dai *et al*.^6^ introduced non-homogenous weighted Koch networks, and defined the mean weighted first-passage time (MWFPT) inspired by the definition of the average weighted shortest path. Sun *et al*.^4^ discussed a family of weighted hierarchical networks which are recursively defined from an initial uncompleted graph. Zhu *et al*.^7^ reported a weighted hierarchical network generated on the basis of self-similarity, and calculated analytically the expression of the MFPTs with weight-dependent walk by using a recursion relation of the hierarchical network structure. Sun *et al*.^8^ obtained the exact scalings of the mean first-passage time (MFPT) with random walks on a family of small-world treelike networks.

For un-weighted networks, calculating the entire mean first-passage time (EMFPT) generally use three methods, i.e., the definition of the EMFPT^8^,^9^, the average shortest path^10^, and Laplacian spectra^11^,^12^. Sun *et al*.^8^ used the definition of the EMFPT for the considered networks to obtain the analytical expressions of the EMFPT and avoided the calculations of the Laplacian spectra.

In this paper, there are two methods to calculate the entire mean weighted first-passage time (EMWFPT), 〈*F*〉_*n*_, for the weighted treelike networks as follows. Method 1 is to get the asymptotic behavior of the EMWFPT directly by the definition of the EMWFPT. Method 2 is to get the asymptotic behavior of the EMWFPT based on the relationship between 〈*F*〉_*n*_ and *λ*_*n*_, i.e, 〈*F*〉_*n*_ = (*N*_*n*_ − 1)*λ*_*n*_, where *N*_*n*_ is the total number of nodes. The obtained consistent results show that Method 2 is entirely feasible. Thus the effective resistance mean exactly the weight between two adjacency nodes for the weighted treelike networks. Our key finding is profound, which can help us to compute the EMWFPT by the weighted Laplacian spectra.

The organization of this paper is as follows. In next section we introduce a family of weighted treelike networks. Then we give the definition of the EMWFPT and use two methods to calculate it. In the last section we draw conclusions.

## Weighted treelike networks

Recently, there are several literatures on the preferential-attachment (scale-free) method of generating a random by adding a very specific way of generating weights^1^,^13^,^14^. Based on Barabasi-Albert model, deterministic networks have attracted increasing attention because they have an advantage with precise formulations on some attributes. In this section a family of weighted treelike networks are introduced^15^,^16^,^17^, which are constructed in a deterministic iterative way. The recursive weighted treelike networks are constructed as follows.

Let *r*(0 < *r* < 1) be a positive real numbers, and *s*(*s* > 1) be a positive integer.

(1) Let *G*_0_ be base graph, with its attaching node *a*_0_ and the other nodes 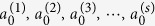. Each node of 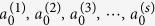 links the attaching node *a*_0_ with unitary weight. We also call the attaching node *a*_0_ as the central node.

(2) For any *n* ≥ 1, *G*_*n*_ is obtained from *G*_*n*−1_: *G*_*n*_ has one attaching node labelled by *a*_*n*_, that we call *a*_*n*_ as the central node of *G*_*n*_. Let 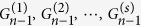 be *s* copies of *G*_*n*−1_, whose weighted edges have been scaled by a weight factor *r*. For 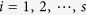, let us denote by 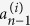 the node in 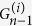 image of 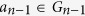, then link all those 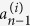 to the attaching node .. through edges of unitary weight. Let *G*_*n*_ = *G*(*V*_*n*_, *E*_*n*_) be its associated weighted treelike network, with vertex set *V*_*n*_(|*V*_*n*_| = *N*_*n*_) and edge set *E*_*n*_(|*E*_*n*_| = *N*_*n*_ − 1). Similarly, 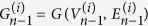, 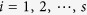. In Fig. 1, we schematically illustrate the process of the first three iterations.

The weighted treelike networks are set up.

According to the construction method of *G*_*n*_ (*n* ≥ 1), *G*_*n*_ can be regarded as merging *s* + 1 groups, sequentially denoted by 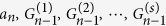. (see Fig. 1).

From the construction of the weighted treelike networks, one can see that *G*_*n*_, the weighted treelike networks of *n*-th generation, is characterized by three parameters *n, s* and *r: n* being the number of generations, *s* being the number of copies, and *r* representing the weight factor. The total number of nodes *N*_*n*_ in *G*_*n*_ satisfy the following relationship, i.e. 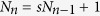. Then


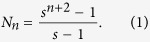


## The entire mean weighted first-passing time

Assuming that the walker, at each step, starting from its current node, moves uniformly to any of its nearest neighbors. For two adjacency nodes *i* and *j*, the weighted time is defined as the corresponding edge weight *w*_*ij*_. The mean weighted first-passing time (MWFPT) is the expected first arriving weighted time for the walks starting from a source node to a given target node. Let the source node be *i* and the given target node be *j*, denote *F*_*ij*_(*n*) by the MWFPT for a walker starting from node *i* to node *j*. We consider here the entire mean weighted first-passage time 〈*F*〉_*n*_ as the average of *F*_*ij*_(*n*) over all pair of vertices,


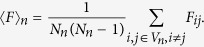


To calculate the asymptotic behavior of the 〈*F*〉_*n*_ for this model, we focus on the two methods, the definition of the EMWFPT and the average shortest path, respectively.

## Method 1

In this section, we compute the EMWFPT using the definition of the EMWFPT. **Step 1**, we study the first quantity *Q*_*tot*_(*n*), i.e, the sum of MWFPTs for all nodes in 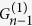 to absorption at the central node *a*_*n*_. **Step 2**, we study the second quantity *H*_*tot*_(*n*), i.e, the sum of MWFPTs for central node *a*_*n*_ to arrive all nodes in 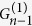 of *G*_*n*_. **Step 3**, we use the definition to obtain the asymptotic behavior of the MWFPT between all node pairs and the asymptotic behavior of the EMWFPT in the limited of large *n*.

### Step 1

We study the first quantity *Q*_*tot*_(*n*), i.e, the sum of MWFPTs for all nodes in 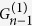 to absorption at the central node *a*_*n*_. Defining 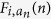 be the MWFPT of a walker from node *i* to the central node *a*_*n*_ for the first time. We denote by *T*_*tot*_(*n*) the sum of MWFPTS for all nodes of *G*_*n*_ to absorption at the central node *a*_*n*_.

We have already arrived the result about *T*_*tot*_(*n*)^15^, i.e.


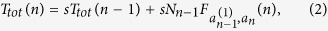


where 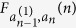 be the MWFPT from Node 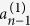 to the central node *a*_*n*_. Thus, the problem of determining *T*_*tot*_(*n*) is reduced to finding 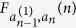. Note that the degree of the node 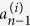 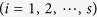 is *s* + 1, we obtain


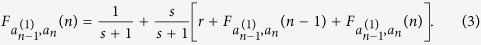


Through the reduction of Eq. (3), we obtain


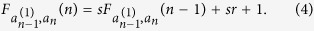


With the initial condition of 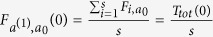, Eq. (4) is inductively solved as


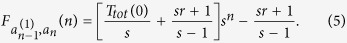


Inserting 
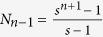
 and Eq. (5) into Eq. (2), we obtain the exact solution of MWFPT from all other nodes to the central node on the networks *G*_*n*_ as follow


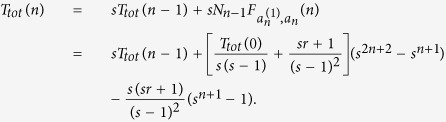


Then,


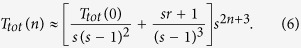


From the definition of *T*_*tot*_(*n*), *T*_*tot*_(*n*) is given by


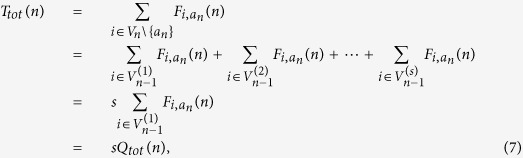


where 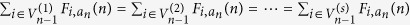. Recalling that Eq. (1), the asymptotic behavior of *Q*_*tot*_(*n*) in the limited of large *n* is as follows,


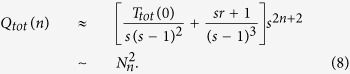


### Step 2

We study the second quantity *H*_*tot*_(*n*), i.e, the sum of MWFPTs for central node *a*_*n*_ to arrive all nodes in 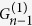 of *G*_*n*_. Firstly, let *R*_*i*_(*n*) denote the expected weighted time for a walker in weighted networks *G*_*n*_, originating from node *i* to return to the starting point *i* for the first time, named mean weighted return time. By definition of 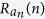, we obtain


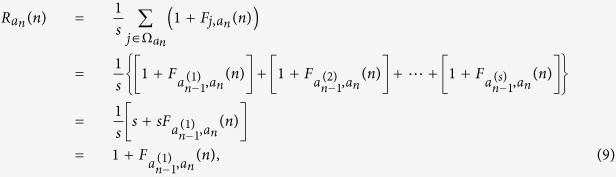


where 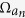 is the set of neighbors of the central node *a*_*n*_ and 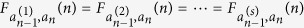. Using Eq. (5) and Eq. (9) is solved as


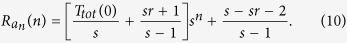


Note that the degree of the node *a*_*n*_ 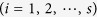 is *s*, we obtain


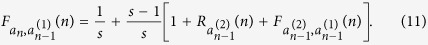


Similarly,


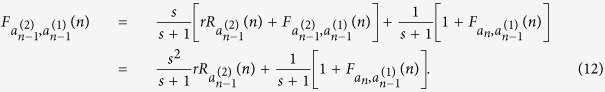


Inserting Eq. (12) into Eq. (11), we obtain


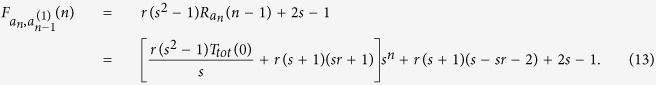


From the definition of *H*_*tot*_(*n*), *H*_*tot*_(*n*) is written by


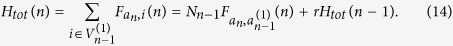


Recalling Eq. (1) and Eq. (13), Eq. (14) is solved as


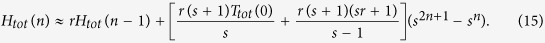


The asymptotic behavior of *H*_*tot*_(*n*) in the limited of large *n* is as follows,


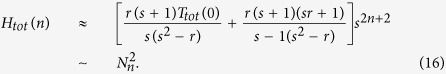


### Step 3

We use the definition to obtain the asymptotic behavior of the EMWFPT in the limited of large *n*. Starting from the definition of the EMWFPT and the recursive construction, we can decompose the *F*_*tot*_(*n*) into four terms:


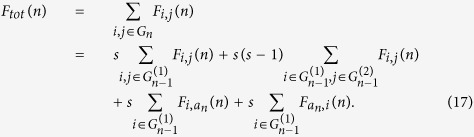


The first term takes into account a walker starting from and arriving at nodes belonging to the same subgraph. The second term takes into account all the possible paths where the initial point and the final one belong to two different subgraphs, and we can set them to 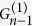 and 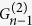 and multiply the contribution by a combinatorial factor *s*(*s* − 1). Finally the last two terms takes into account all the possible paths between each of nodes of subgraph 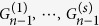 and the central node *a*_*n*_.

Using the scaling mechanism for the edges, the first term in Eq. (17) can be easily identified with


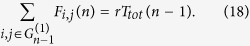


By construction, each pass connecting two nodes belonging to two different subgraphs, must pass through the central node *a*_*n*_, hence using 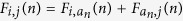, the second term of Eq. (17) can be split into two parts:


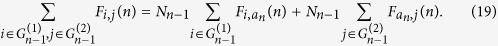


Then, Eq. (17) can be simplified as


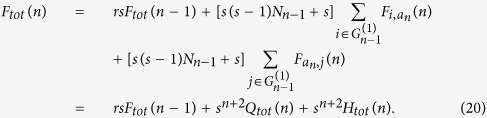


Inserting Eq. (8) and Eq. (16) into Eq. (20), the asymptotic behavior of *F*_*tot*_(*n*) in the limited of large *n* is as follows,


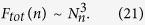


and


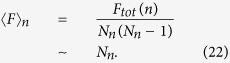


## Method 2

In this section, Method 2 is that the average weighted shortest path used to get the asymptotic behavior the EMWFPT. This method gives some significantly new insights more straightforward than Method 1.

The resistance distance *r*_*ij*_ between two nodes *i* and *j* is defined as the effective (electrical) resistance between them when each weighted edge has been replaced by a resistor. It is known that the weighted first-passage time between two nodes is related to their resistance distance by 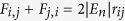
18,19 and, in which 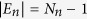 is the total number of edges for weighted treelike network *G*_*n*_ and *F*_*i*,*j*_ = *F*_*j*,*i*_. The EMWFPT of weighted treelike network is


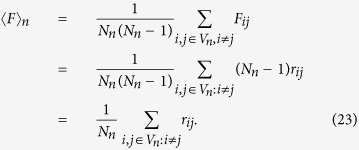


Let *λ*_*ij*_ as the weighted shortest path between two nodes *i* and *j* of the weighted networks *G*_*n*_^2^. For any weighted treelike networks, the weighted shortest path *λ*_*ij*_ of *G*_*n*_ is equal to the effective resistance *r*_*ij*_ between node *i* and *j*, i.e, *r*_*ij*_ = *λ*_*ij*_. By definition the average weighted shortest path *λ*_*n*_ of the graph *G*_*n*_ is given by^4^


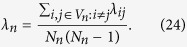


For a large system, i.e., *N*_*n*_ → ∞, we have already known that the *λ*_*n*_ of the *G*_*n*_ is (see ref. 17).


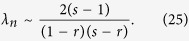


Now we substitute Eq. (24) and Eq. (25) into Eq. (23) obtaining,


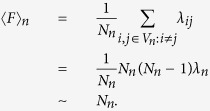


This result coincides with the asymptotic behavior 〈*F*〉_*n*_ in Eq. (22). Therefore, we can draw the conclusion that the effective resistance mean exactly the weight between two adjacency nodes for the weighted treelike networks.

## Conclusions

In this paper, we have proposed a family of weighted treelike networks formed by three parameters as a generalization of the un-weighted trees. We have calculated the entire mean weighted first-passage time (EMWFPT) with random walks on a family of weighted treelike networks. We have used two methods to obtain the asymptotic behavior of the EMWFPT with regard to network parameters. Firstly, using the construction algorithm, we have calculated the receiving and sending times from the central nodes to the other nodes of 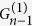 to obtain the asymptotic behavior of the EMWFPT. Secondly, applying the relationship equation between EMWFPT and the average weighted shortest path, we also have obtained the asymptotic behavior of the EMWFPT. The dominating terms of the EMWFPT obtained by two methods are coincident, which shows that the effective resistance is equal to the weight between two adjacency nodes. Noticed that the dominating term of the EMWFPT scales linearly with network size *N*_*n*_ in large network. It is expected that the edge-weighted adjacency matrices can be used to compute the weighted Laplacian spectra to obtain the asymptotic behavior of the EMWFPT of weighted treelike networks.

## Additional Information

**How to cite this article**: Dai, M. *et al*. The entire mean weighted first-passage time on a family of weighted treelike networks. *Sci. Rep.* **6**, 28733; doi: 10.1038/srep28733 (2016).

## Figures and Tables

**Figure 1:**